# *Listeria monocytogenes* Infection in Israel and Review of Cases Worldwide

**DOI:** 10.3201/eid0803.010195

**Published:** 2002-03

**Authors:** Yardena Siegman-Igra, Rotem Levin, Miriam Weinberger, Yoav Golan, David Schwartz, Zmira Samra, Hana Konigsberger, Amos Yinnon, Galia Rahav, Nathan Keller, Nail Bisharat, Jehuda Karpuch, Renato Finkelstein, Michael Alkan, Zvi Landau, Julia Novikov, David Hassin, Carlos Rudnicki, Ruth Kitzes, Shmouel Ovadia, Zvi Shimoni, Ruth Lang, Tamar Shohat

**Affiliations:** * Sourasky, Tel-Aviv, Israel; † Rabin, Petach Tikva, Israel; ‡ Shaare-Zedek, Jerusalem, Israel; § Hadassah, Jerusalem, Israel; ¶ Sheba, Tel-Hashomer, Israel; # Ha’Emek, Afula, Israel; ** Assaf-Harofeh, Zriffin, Israel; †† Rambam, Haifa, Israel; ‡‡ Soroka, Beer-Sheva, Israel; §§ Kaplan, Rechovot, Israel; ¶¶ Bnei-Zion, Haifa, Israel; ## Hillel-Yaffe, Hadera, Israel; *** Carmel, Haifa, Israel; ††† Wolfson, Holon, Israel; ‡‡‡ Laniado, Netanya, Israel; §§§ Sapir, Kfar-Saba, Israel; ¶¶¶ District Health Office, Ministry of Health, Tel-Aviv, Israel

**Keywords:** *Listeria monocytogenes*, listeriosis, epidemiology, neonatal infection, foodborne disease, immuonocompromised hosts

## Abstract

*Listeria monocytogenes*, an uncommon foodborne pathogen, is increasingly recognized as a cause of life-threatening disease. A marked increase in reported cases of listeriosis during 1998 motivated a retrospective nationwide survey of the infection in Israel. From 1995 to 1999, 161 cases were identified; 70 (43%) were perinatal infections, with a fetal mortality rate of 45%. Most (74%) of the 91 nonperinatal infections involved immunocompromised patients with malignancies, chronic liver disease, chronic renal failure, or diabetes mellitus. The common clinical syndromes in these patients were primary bacteremia (47%) and meningitis (28%). The crude case-fatality rate in this group was 38%, with a higher death rate in immunocompromised patients.

*Listeria monocytogenes* (Lm) is a ubiquitous pathogen in the environment, capable of causing human and animal infection. Although uncommon in humans, it occurs in sporadic and epidemic forms throughout the world (1–3); a recent multistate outbreak was reported in the United States (4). Most and perhaps all forms of listeriosis in humans result from foodborne transmission (5). In its most severe form, listeriosis is an invasive disease that affects immunocompromised patients and has the highest case-fatality rate of foodborne illnesses (6–10). In immunocompetent persons, it can also cause severe disease (attributed by some investigators to ingestion of high infective doses), as well as outbreaks of benign febrile gastroenteritis (11). Another form of human disease is perinatal infection, which is associated with a high rate of fetal loss (including full-term stillbirths) and serious neonatal disease (12).

Lm infection has been a reportable disease in Israel since 1993. A preliminary report from the Ministry of Health (MOH) claimed a fivefold increase in incidence from 1996 to 1998, but the information was incomplete (13). Our study was undertaken to delineate trends and better characterize the epidemiologic and clinical features of this emerging infection in Israel and to compare these findings with those reported in recent publications worldwide.

## Material and Methods

### The Israeli Survey

Of the 24 general (acute-care) hospitals in Israel, 11 are large, with 500-1,200 beds, 8 have 300-499 beds, and 5 have <300 beds. Information on Lm infections was collected by contacting infectious disease specialists in each of the 19 larger hospitals. The specialists were asked to identify retrospectively all patients with listeriosis (as defined below) from the period 1995-1999 in their hospitals and to complete a questionnaire on each. Questionnaires were completed from 17 of the hospitals (11 large, 6 intermediate), and complementary information was retrieved from the MOH passive and active surveillance files on 4 additional hospitals (1 intermediate and 3 small). These 21 hospitals represented approximately 95% of the total acute-care beds in Israel during the study period.

One hundred sixty-one patients with Lm infection were identified. Clinical information was available for all patients except five (3%: two with positive blood cultures and one with a positive vaginal culture who were not hospitalized, and two with positive blood cultures whose hospital charts could not be retrieved).

Lm isolates were identified by standard methods in the microbiology laboratory in each medical center, then sent to the Reference Laboratory for Listeria in Jerusalem for confirmation.

Listeriosis was defined as the growth of Lm (as confirmed at the reference laboratory) from any body site. An infection in a pregnant woman and her fetus or neonate was considered a single perinatal event.

### Worldwide Review

We conducted aPubmed search for studies describing nonselective, population-based surveys of Lm infections in the English language literature of the last decade (1990-2000). All case series describing at least 15 nonperinatal, nongastroenteritis infections were included in the review of nonperinatal listeriosis. All case series describing at least 15 perinatal cases were included in the review of perinatal listeriosis.

## Results

### The Israeli Survey (1995-1999)

**Figure 1:**
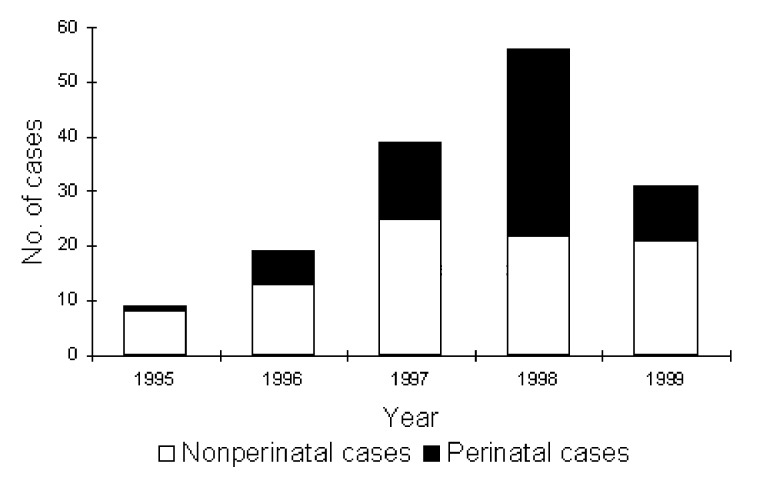
Number of cases of perinatal and nonperinatal *Listeria monocytogenes* infection, Israel, 1995-1999.

**Figure 2:**
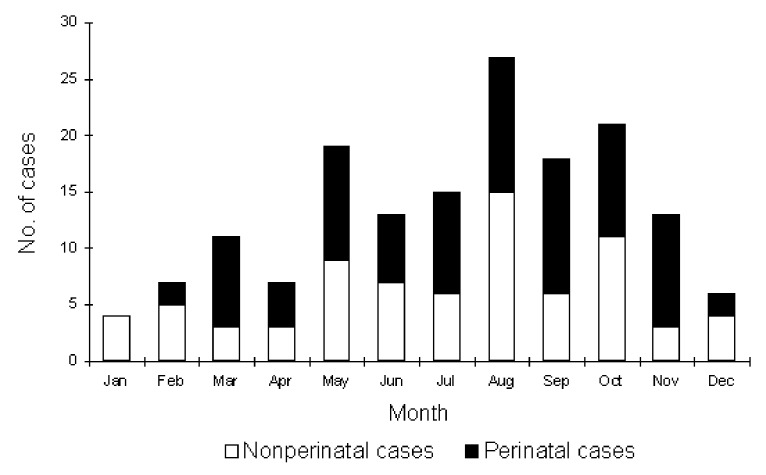
Seasonal occurrence of *Listeria monocytogenes* infection, Israel, 1995-1999.

The 161 cases identified during the 5-year study period included 91 (57%) nonperinatal and 70 (43%) perinatal infections. The average annual incidence during the study period was 0.6/100,000 population. The marked increase in 1998 (Figure 1) was exclusively in perinatal cases; the reason for the increase remains unclear. There were no clusters in place during any of these years. Infection occurred throughout the year, but more often during summer and fall, with 70% of cases occurring from May to October (Figure 2).

#### <H3>Nonperinatal Cases

**Figure 3:**
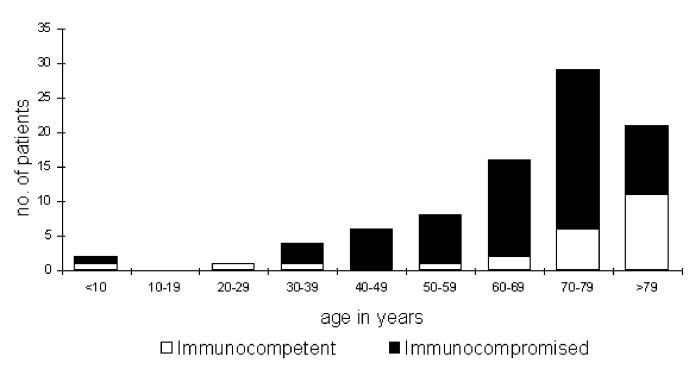
Age distribution of 87 nonperinatal cases of *Listeria monocytogenes* infection by immune-status group, Israel, 1995-1999.

The mean age of the 87 nonperinatal cases with available clinical information was 67 years (range 4-91), 66 (76%) were ≥60 years of age (Figure 3); 56 (64%) were male. Sixty-four patients (74%) had severe immunocompromising conditions (Table 1). Of 45 patients (52%) with malignant disease, most had received chemotherapy, steroid therapy, or both during the month before the Lm infection. Other immunocompromising conditions were chronic renal failure (11 patients, 4 of whom were on hemodialysis), chronic liver disease (10 patients, mostly with cirrhosis), and diabetes mellitus (13 patients). Some of these patients had additional immunocompromising conditions (Table 1). Twenty-three patients (26%) were not immunocompromised. Most (19 [83%] of 23) were ≥63 years of age; some had concomitant conditions not considered to be immunocompromising, including three patients with valvular heart disease, predisposing them to endocarditis. Only four immunocompetent patients were <60 years of age, including a 4-year-old girl and a 38-year-old man with primary bacteremia, a 22-year-old woman with typical pyelonephritis and Lm cultured from blood only, and a 51-year-old man who had gastroenteritis and positive blood cultures (stool was not cultured for Lm).

**Table 1:** Immunocompromising conditions in 64 cases of non-perinatal *Listeria monocytogenes* infection, Israel, 1995-1999

Main underlying illness	No. of cases	Additional underlying conditions				
		Steroids/ chemotherapy	Chronic renal failure	Chronic liver disease	Diabetes mellitus	Others
Hematologic malignancy	23	19	3	2	7	8^a^
Solid malignancy	22	9				
Chronic renal failure	8^b^	1			2	1^c^
Chronic liver disease	8	1			1	2^c^
Diabetes mellitus	3	1				

^a^Splenectomy (2 cases) neutropenia (5) vasculitis (1). ^b^Hemodialysis (4 cases). ^c^Severe congestive heart failure (3 cases).

Clinical syndromes in the 87 nonperinatal cases were primary bacteremia in 41 (47%), meningitis in 24 (28%), bacteremia with a focal infection in 18 (21%), and focal infection without bacteremia in 4 (5%) (Table 2). Six patients with meningitis had Lm isolated from both blood and cerebrospinal fluid (CSF). Two patients with primary bacteremia had symptoms suggestive of meningitis (confusion, aggressiveness), but they died shortly after admission without having a lumbar puncture performed.

**Table 2:** Clinical syndromes in 87 cases of nonperinatal *Listeria monocytogenes* infection, Israel, 1995-99

Clinical syndrome	Immunocompromised	Immunocompetent	Total
Bacteremia without focus	34 (53%)	7 (30%)	41 (47%)
Meningitis	17^a^ (27%)	7 (30%)	24 (28%)
Bacteremia with focus	9^b^ (14%)	9^c^ (39%)	18 (21%)
A focus without bacteremia	4^d^ (6%)	0	4 (5%)
Total	64 (100%)	23 (100%)	87 (100%)

^a^ With bacteremia (6 cases), with pneumonia (1 case). ^b^ Endocarditis (3 cases), peritonitis (2), pneumonia (4, one with shunt infection). ^c^ Endocarditis (3 cases), gastroenteritis (3), pyelonephritis (2), anal abscess (1). ^d^ Peritonitis (3 cases), pleuritis (1).

The case-fatality rate in the nonperinatal group was 38% (33 of 87). Twelve of the 33 deaths occurred within 48 hours of admission or disease onset. We observed a lower mortality rate (6 [19%] of 31) among persons who received a penicillin (mostly ampicillin) as empiric therapy, compared with those who received a penicillin only after culture results were reported (9 [30%] of 30), but this difference was not statistically significant (p=0.25). The difference in death rates in immunocompromised (28 [44%] of 64) compared with immunocompetent patients (5 [22%] of 23) had borderline statistical significance (p=0.05). There was no correlation between death and age for the whole group; however, all five immunocompetent patients who died were >80 years of age.

#### <H3>Perinatal Cases

Clinical information was available on 69 pregnant women (mean age 28 years; range 21-40 years) and their offspring. Twenty-seven pregnancies (gestational age 9-26 weeks) resulted in intrauterine fetal death and miscarriage, one full-term infant was stillborn, and three others (born at 26, 29, and 39 weeks) died within 24-48 hours of birth (Table 3), for a mortality rate among offspring of 45%. Seventeen (55%) of the 31 infected mothers whose offspring died were bacteremic. For the other 14 mothers, Lm was isolated from other sites, including placenta, amniotic fluid, and fetal tissue (Table 3).

**Table 3:** Types of infection, sources of cultures, and outcome in 69 cases of perinatal *Listeria monocytogenes* infection, Israel, 1995-99

		Mothers’ cultures			Infants’ cultures		
Type of mother-infant infection	No. of cases	Blood only	Blood and tissue	Tissue only	Blood only	Blood and tissue	Tissue only
Uninfected mother and infected infant	13 (19%)				4	7 (3)	2 (2)
Infected mother and infected infant	9 (13%)	2	1	6	3	2	4 (1)
Infected mother and uninfected infant	16 (23%)	9	3	4			
Fetal/neonatal death (amnionitis)	31^a^ (45%)	4	13	14	1	1 (1)	
Total	69 (100%)	54			24		

^a^Includes 27 intrauterine deaths with abortions, 1 stillbirth, and 3 early neonatal deaths. Numbers in parenthesis are cases of meningitis. Tissue denotes any material that is not blood, such as cerebrospinal fluid, placenta, and amniotic fluid.

The other 38 mothers gave birth to live infants, 16 of whom had no evidence of Lm infection. Eleven of the 16 uninfected infants were delivered when the mothers had active Lm amnionitis (gestational ages 25 to 40 weeks), and 5 were delivered several weeks after the maternal infection, which occurred at weeks 19, 21, 35, 36, and 37. Twenty-two infants had evidence of Lm infection after birth, 18 within 48 hours of delivery and 4 on days 4-8. Only two (11%) of the 18 infants with early infection had meningitis, compared with all 4 with later onset of infection.

All infected mothers had mild illness and recovered uneventfully; none had meningitis. One mother had an underlying immunocompromising condition (systemic lupus erythematosus).

### Worldwide Review (1990-2000)

Nine case-series of nonperinatal listeriosis and five case-series of perinatal infection matched the inclusion criteria. These reports and our study provided 1,250 cases of nonperinatal and 494 cases of perinatal listeriosis for analysis (Tables 4 and 5). Nonperinatal infection constituted, on average, 65% (1,025 of 1,583) of cases among studies that supplied this information (Table 4). In total, 1,250 cases of nonperinatal infections were reviewed; information about mortality was provided for 1,129 patients. The patients’ ages ranged from <1 year to >90 years, but most were >60 years of age. The mean ages in the different series ranged from 50 to 67 years; 60% were male. Annual incidence rate varied widely (i.e., 0.1-1.1 per 10^5^), not only between countries but also between consecutive years in the same setting. Most authors also described seasonal variation, with a peak incidence in summer possibly related to seasonal consumption of specific food products (8) or to more frequent breakdowns in food handling in higher temperatures.

**Table 4:** Characteristics of nonperinatal listeriosis from 10 recently reported series

	First author, year (ref)										
Characteristic	McLauchlin, 1990 (8)	Gellin, 1991 (6)	Cherubin, 1991 (14)	Skogberg, 1992 (15)	Nolla-Salas, 1993 (16)	Jones, 1994 (17)	Paul, 1994 (18)	Bula, 1995 (10)	Goulet, 1996 (9)	Siegman-Igra, 2001 (present study)	Total or average
Country and scope	England, national	USA, six areas	USA, four centers	Finland, Helsinki	Spain, Barcelona	England, Bristol	Australia, Sydney	Switz-erland, western part	France, national	Israel, national	Worldwide
Study period	1967-1985	1986	1982-1999	1971-1989	1990	1983-1992	1983-1992	1983-1997	1992	1995-1999	1967-1999
Total no. of cases	722	246	119	74	31	29	84	122	225^a^	156	1,808
Nonperinatal cases (% of total)	474 (66%)	179 (73%)	54 (45%)	58 (78%)	29 (94%)	16 (55%)	71 (85%)	57 (47%)	225 (NA)	87 (56%)	1,025/1583 (65%) 1,025+225=1,250
Mean age (range) (years)	59 (1-97)	NA (<1-95)	NA	50 (29-66)	58 (17-89)	60 (1-95)	39% (>60)	66 (31-96)	65 (1-101)	67 (4-91)	50-67
Male gender	265 (58%)	101 (56%)	NA	NA	24 (77%)	9 (56%)	NA	33 (58%)	135 (62%)	56 (64%)	623 (60%)
Peak season	Autumn and spring	Late spring to fall	May-Aug	NA	39% in July-Sept	76% in July-Dec	NA	NA	NA	70% in May-Oct	Summer and fall
Annual incidence^b^	NA	0.7	NA	0.09 - 0.65	1.1	0.35	0.3	NA	NA	0.6	0.1-1.1
Immunocompromised	261/337 (77%)	NA	53/54(98%)	47/58(81%)	24/29 (83%)	13/16 (81%)	59/71 (83%)	25/57 (42%)	159/225(71%)	64/87 (74%)	705/934 (74%)
CNS infection	268/474 (57%)	55/179 (31%)	19/54 (35%)	29/58 (50%)	9/31 (29%)	6/16 (37%)	29/71 (41%)	45/57 (79%)	110/224(49%)	24/87 (28%)	594/1,251 (47%)
Bacteremia + focus^c^	183/474 (39%)	119/179 (66%)	35/54 (65%)	24/58 (41%)	20/31 (65%)	10/16 (73%)	40/71 (56%)	12/57 (21%)	97/224 (43%)	59/87 (68%)	599/1,251 (48%)
Focal disease only^d^	9/474 (5%)	5/179 (3%)	-	5/58 (8%)	2/31 (6%)	-	2/71 (3%)	-	17/224 (8%)	4/87 (5%)	44/1,124 (4%)
Mortality	164/371 (44%)	63/179(35%)	17/54(31%)	15/58(26%)	16/31 (52%)	6/16 (37%)	27/71 (38%)	18/57 (32%)	54/225 (24%)	33/87 (38%)	413/1,149 (36%)

^a^Includes nonperinatal cases only. ^b^Estimated annual incidence per 10^5^ population. ^c^Bacteremia with or without a non-CNS focus of infection (e.g., pneumonia, endocarditis, urinary tract infection, prostatitis, peritonitis, gastroenteritis, rectal abscess, osteomyelitis, and cellulitis). ^d^For example, peritonitis, pleuritis, arthritis, pericarditis, cholecystitis, appendicitis, and cellulitis. NA= not available; CNS= central nervous system.

**Table 5:** Characteristics of perinatal listeriosis from six recently reported series

	First author, year (ref)						
Characteristic	McLauchlin, 1990 (12)	Gellin, 1991 (6)	Cherubin, 1991 (14)	Craig, 1996 (19)	Nolla-Salas, 1998 (20)	Siegman-Igra, 2001 (present study)	Total or average
Country and region	England, national	USA, six areas	USA, four centers	Australia, Melbourne	Spain, Barcelona	Israel, national	Worldwide
Study period	1967-1985	1986	1982-1999	1983-1994	1990-1996	1995-1999	1967-1999
Total no. of cases	722	246	119	24^a^	135	156	1,400
Perinatal infection (% of total)	248 (34%)	67 (27%)	65 (55%)	24 (NA)	21 (16%)	69 (44%)	470/1,378 (34%) 470+24=494
Estimated incidence per 10^4^ births	NA	0.8-2.4	NA	2	0-4.1	1.4	0.6-4.1
Average maternal age (range) (years)	NA	26 (17-35)	NA	NA (18-39)	30 (26-34)	28 (21-40)	NA (26-30)
Early neonatal infection and survival	114^b^ (46%)	31 (46%)	20 (31%)	14^c^ (58%)	11 (52%)	19 (28%)	209/494 (42%)
Late neonatal infection and survival	36^d^ (15%)	8^e^ (12%)	21(32%)		1^d^ (5%)	3^d^ (4%)	69/494 (14%)
Infected mother and uninfected infant	9 (4%)	13 (19%)	2 (3%)	4 (17%)	5 (23%)	16 (23%)	49/494 (10%)
Intrauterine death	42 (17%)	14 (21%)	15 (23%)	4 (17%)	3 (14%)	28 (41%)	106/494 (21%)
Postnatal death	47 (19%)	1 (1%)	7 (11%)	2 (8%)	1 (5%)	3 (4%)	61/494 (12%)
Gestational age at abortion (weeks)	12-28	11-30	NA	18-29	10-27	9-26	9-29
Immunocompromised mothers	5			1	1	1	8^f^

^a^Includes only perinatal cases. ^b^Including 29 with unknown time of onset. ^c^No differentiation between early and late neonatal infection. ^d^>5 days. ^e^>7 days. ^f^2 diabetes mellitus, 2 renal transplant, 2 systemic lupus erythematosus, 1 Crohn disease and steroids, 1 HIV infection. NA= not available

Most (74%) of the persons affected in the reported cases (Table 4) were immunocompromised. Malignancy, chemotherapy, steroid therapy, organ transplantation, alcoholism, liver disease, renal insufficiency, and diabetes mellitus were most commonly reported, with few cases of acquired immunodeficiency syndrome.

With regard to clinical syndromes, the most common (47%) site of infection was the central nervous system (CNS) (Table 4), frequently associated with bacteremia. An additional 48% of patients were bacteremic without CNS involvement.

Perinatal infection constituted 34% (470 of 1,378) of cases among studies that supplied this information (Table 5). In total, 494 cases of perinatal infections were reviewed. Infection during the first two trimesters of pregnancy was almost invariably fatal to the fetus. One hundred six (21%) of the 494 pregnancies reviewed here resulted in intrauterine death.

Two hundred seventy-eight (56%) live-born infants had neonatal listeriosis and survived. Most of this neonatal infection was of early onset (209 cases), but the definition of early onset varied (from ≤5 to ≤7 days), and information concerning day of onset was incomplete in some series (Table 5). Almost all the late-onset infections (69 cases) were of the CNS. An additional 61 infants (12%) with neonatal listeriosis died from the infection in the postnatal period, for an overall intrauterine and postnatal mortality rate of 34% (167 of 494). In 49 (10%) of the affected pregnancies, the infant was born alive and without evidence of listeriosis.

## Discussion

Ingestion of Lm is a very common occurrence (1,2) since it has been isolated from many food products in Israel (unpub. data, MOH) as well as in many countries worldwide. Development of invasive disease secondary to Lm ingestion is determined primarily by the integrity of the immune system of the host (predominantly cell-mediated immune defects) and possibly also by inoculum size (11). The organism crosses the mucosal barrier of the intestine and invades the bloodstream. It may disseminate to any organ, but it has a clear predilection for the placenta and CNS, thereby determining the main clinical syndromes.

The case-fatality rate in the collected data on perinatal infection was 36% (413 of 1,149 patients for whom this information was available). This high mortality reflects both the severity of Lm infection and the seriousness of the underlying conditions. Higher mortality rates were correlated with older age, presence of CNS infection, and immunodeficiency (5,6,8,15,21). One study reported that deaths in immunocompetent patients occurred exclusively in the elderly (9), a finding that correlates well with our observations.

An unexpected observation in our study was the occurrence of hospital-acquired listeriosis in adults. The presumed hospital acquisition occurred on day 3-67 of hospital stay in 59 (16%) of 369 cases with relevant information, as reported in four studies, including ours (9,16,18). All patients acquiring listeriosis in the hospital (except one) were immunocompromised. No clustering of cases in time or place occurred, and no case had an obvious source for nosocomial acquisition of Lm. Because the incubation period of listeriosis is long (11-70 days) and fecal carriage not uncommon (5%-10%) (1,2), colonization could have been acquired before hospitalization and infection developed in the hospital, possibly even triggered by increased immunosuppression. Another possible explanation is consumption of contaminated food brought in from sources outside the hospital, but this could not be documented. We found only one description of a hospital outbreak of Lm among adults (three cases secondary to an index one), but the method of transmission was not established (22). Hospital transmission among neonates in nurseries was thought to occur more frequently (24%) (12) and was described by several investigators (18,23,24).

Among perinatal infections, we report the highest case-fatality rate (45%). This observation could be related to the frequency of taking cultures from aborted tissues. The diagnosis of Lm can easily be missed if cultures are not routinely taken from aborted fetal tissues or if blood (and other) cultures are not obtained from febrile pregnant women. The great variability in incidence rates and other epidemiologic features between studies and among medical centers within studies suggests that many cases escaped diagnosis.

Concerning the mothers, all authors described a mild febrile “influenzalike” illness, without maternal deaths. Only one of the 494 mothers had meningoencephalitis with Lm isolated from the cerebrospinal fluid, but underlying condition or maternal and fetal outcomes were not reported (12). Eight mothers (<2%) were immunocompromised (Table 5), but no comparable data are available on the prevalence of these conditions among pregnant women in general.

In conclusion, listeriosis is an emerging zoonosis that constitutes a life-threatening disease for human fetuses and neonates, the elderly, and patients with certain predisposing conditions. Documented cases may not represent the true incidence in the community, especially with regard to perinatal infection. Fetal and maternal cultures should be obtained in every case of spontaneous abortion or stillbirth, to ensure proper diagnosis. Empiric ampicillin therapy should be included in the treatment of neonatal meningitis, sepsis, or meningitis in the elderly and immunocompromised patients and in febrile pregnant women without a source of infection.

## References

[1] Listeriosis LB. Clin Infect Dis. 1997;24:1–11. 8994747 10.1093/clinids/24.1.1

[2] Farber JM, Peterkin PI. Listeria monocytogenes, A food-borne pathogen. Microbiol Rev. 1991;55:476–511. 1943998 10.1128/mr.55.3.476-511.1991 PMC372831

[3] Schlech WF. Foodborne listeriosis. Clin Infect Dis. 2000;31:770–5. 10.1086/314008 11017828

[4] Centers for Disease Control and Prevention. Multistate outbreak of Listeriosis—United States, 2000. MMWR Morb Mortal Wkly Rep. 2000;49:1129–30. 11190115

[5] Schuchat A, Deaver KA, Wenger JD, Plikaytis BD, Mascola L, Pinner RW, Role of foods in sporadic listeriosis I. Case-control study of dietary risk factors. JAMA. 1992;267:2041–5. 10.1001/jama.267.15.2041 1552639

[6] Gellin BG, Broome CV, Bibb WF, Weaver RE, Gaventa S, Mascola L, ; Listeriosis Study Group. The epidemiology of listeriosis in the United States-1986. Am J Epidemiol. 1991;133:392–401. 1899779 10.1093/oxfordjournals.aje.a115893

[7] Louria DB, Blevins A, Armstrong D. Listeria infections. Ann N Y Acad Sci. 1970;174:545–51. 10.1111/j.1749-6632.1970.tb45580.x 4993531

[8] McLauchlin J. Human listeriosis in Britain, 1967-85, a summary of 722 cases. 2. Listeriosis in nonpregnant individuals, a changing pattern of infection and seasonal incidence. Epidemiol Infect. 1990;104:191–201. 2108870 10.1017/s0950268800059355 PMC2271748

[9] Goulet V, Marchetti P. Listeriosis in 225 non-pregnant patients in 1992: clinical aspects and outcome in relation to predisposing conditions. Scand J Infect Dis. 1996;28:367–74. 10.3109/00365549609037921 8893400

[10] Bula CJ, Bille J, Glauser MP. An epidemic of food-borne listeriosis in Western Switzerland: description of 57 cases involving adults. Clin Infect Dis. 1995;20:66–72. 7727673 10.1093/clinids/20.1.66

[11] Aureli P, Fiorucci GC, Caroli D, Marchiaro G, Novara O, Leone L, An outbreak of febrile gastroenteritis associated with corn contaminated by *Listeria monocytogenes.* N Engl J Med. 2000;342:1236–41. 10.1056/NEJM200004273421702 10781619

[12] McLauchlin J. Human listeriosis in Britain, 1967-85, a summary of 722 cases.1. Listeriosis during pregnancy and in the newborn. Epidemiol Infect. 1990;104:181–90. 2108869 10.1017/s0950268800059343 PMC2271760

[13] Mates A, Shohat T, Vasilev V, Agmon V, Hirt R, Igra Y, Human listeriosis in Israel for the period 1996-98. Proceedings of the Annual Meeting of the Israel Society for Microbiology; Jan 31-Feb 1, 2000; Haifa, Israel. ISM News 2000;41:17.

[14] Cherubin CE, Appleman MD, Heseltine PNR, Khayr W, Stratton CW. Epidemiological spectrum and current treatment of listeriosis. Rev Infect Dis. 1991;13:1108–14. 1775844 10.1093/clinids/13.6.1108

[15] Skogberg K, Syrjanen J, Jahkola M, Renkonen OV, Paavonen J, Ahonen J, Clinical presentation and outcome of listeriosis in patients with and without immunosuppresive therapy. Clin Infect Dis. 1992;14:815–21. 1341415 10.1093/clinids/14.4.815

[16] Nolla-Salas J, Anto JM, Almela M, Renkonen OV, Paavonen J, Ahonen J. Incidence of listeriosis in Barcelona, Spain, in 1990. The Collaborative Study Group of Listeriosis of Barcelona. Eur J Clin Microbiol Infect Dis. 1993;12:157–61. 10.1007/BF01967105 8508813

[17] Jones EM, McCulloch SY, Reeves DS, MacGowan AP. A 10 year survey of the epidemiology and clinical aspects of listeriosis in a provincial English city. J Infect. 1994;29:91–103. 10.1016/S0163-4453(94)95249-3 7963642

[18] Paul ML, Dwyer DE, Chow C, Robson J, Chambers I, Eagles G, Listeriosis: a review of eighty-four cases. Med J Aust. 1994;160:389–93. 8170424

[19] Craig S, Permezel M, Doyle L, Mildenhall L, Garland S. Perinatal infection with *Listeria monocytogenes.* Aust N Z J Obstet Gynaecol. 1996;36:286–90. 10.1111/j.1479-828X.1996.tb02712.x 8883752

[20] Nolla-Salas J, Bosch J, Gasser I, Vinas L, de Simon M, Almela M, Perinatal listeriosis: a population-based multicenter study in Barcelona, Spain (1990-1996). Am J Perinatol. 1998;15:461–7. 10.1055/s-2007-994067 9788644

[21] Mylonakis E, Hohmann EL, Calderwood SB. Central nervous system infection with *Listeria monocytogenes.* Medicine (Baltimore). 1998;77:313–36. 10.1097/00005792-199809000-00002 9772921

[22] Green HT, Macaulay MB. Hospital outbreak of Listeria monocytogenes septicemia: a problem of cross infection? Lancet. 1978;2:1039–40. 10.1016/S0140-6736(78)92352-8 82046

[23] Nelson KE. Warren d, Tomasi AM, Raju TN, Vidyasagar D. Transmission of neonatal listeriosis in a delivery room. Am J Dis Child. 1985;139:903–5. 4036924 10.1001/archpedi.1985.02140110057029

[24] Simmons MD, Cockcroft PM, Okubadejo OA. Neonatal listeriosis due to cross-infection in an obstetric theatre. J Infect. 1986;13:235–9. 10.1016/S0163-4453(86)91124-2 3098856

