# Correction Vol. 8, No. 3

**DOI:** 10.3201/eid0806.C10806

**Published:** 2002-06

**Keywords:** errata, erratum, correction

In* Listeria monocytogenes* Infection in Israel and Review of Cases Worldwide, by Y. Siegman-Igra et al., an error appears in the discussion section. The corrected sentence appears below and online at http://wwwnc.cdc.gov/eid/article/8/3/01-0195_article.htm

The case-fatality rate in the collected data on nonperinatal infection was 36% (413 of 1,149 patients for whom this information was available).

We regret any confusion this error may have caused.

